# Protecting patient privacy in tabular synthetic health data: a regulatory perspective

**DOI:** 10.1038/s41746-025-02112-0

**Published:** 2025-11-28

**Authors:** Lisa Pilgram, Haksoo Ko, Adeline Tung, Khaled El Emam

**Affiliations:** 1 School of Epidemiology and Public Health, University of Ottawa, Ottawa, ON Canada; 2 Children’s Hospital of Eastern Ontario Research Institute, Ottawa, ON Canada; 3 Department of Nephrology and Medical Intensive Care, Charité - Universitaetsmedizin Berlin, Berlin, Germany; 4 Seoul National University, Seoul, South Korea; 5 Personal Data Protection Commission, Singapore, Singapore

**Keywords:** Health policy, Medical ethics

## Abstract

Synthetic tabular data generation (SDG) is increasingly important in healthcare research and innovation while preserving patients’ privacy. However, ethical concerns remain, primarily over residual privacy vulnerability and insufficient oversight. This review analyzes the only published SDG regulatory guidelines to date, from United Kingdom, Singapore, and South Korea. All emphasize privacy, acknowledging synthetic data is not inherently free from disclosure risks. Thresholds for sufficiently low risk are yet to be determined.

## Introduction

Synthetic tabular data generation is increasingly gaining attention across multiple sectors, such as healthcare and finance. Synthetic data generation (SDG) describes the process of generating artificial or fake data that can serve as a proxy for real data. An SDG model thereby learns the data distribution and generates new samples from this learned distribution. This training of the SDG model can be informed by domain knowledge, published summary statistics and risk calculators^1–5^. Alternatively, the training of an SDG model can be based on individual-level tabular data whereby the model captures the patterns in that data during model training, and this is the focus of the current article^6^.

Such synthetic data mimics the statistical properties of the underlying training data. Similar to prediction models, the goal during training is to develop an SDG model that generalizes well rather than overfitting to the real training data. This means that it should not be too tailored to the training data. Most models therefore have some integrated regularization mechanisms to prevent overfitting and ensure better generalization.

There are multiple use cases for synthetic data that have emerged, such as de-biasing and data augmentation^7^. The particular use case that is of interest in our analysis is privacy-preserving data sharing^8^, whereby SDG is treated as one type of privacy-enhancing technology (PET) and synthetic data would consequently be considered as a representation of the original data with less privacy risk. The idea of SDG as a PET is more than thirty years old^9^ but gained attention recently as Artificial Intelligence/Machine Learning (AI/ML) models continue to improve and the perceptions of weaknesses in traditional data anonymization methods have grown^10–12^. The privacy-preserving characteristics of SDG follow from there being no one-to-one mapping between the synthetic records that are generated and the real records in the training data (and real individuals).

Recently, some authors have noted that SDG represents a “widening legal loophole” giving free rein to insurance and technology companies to disseminate and reuse synthetic data with no constraints, even deliberately leveraging synthetic data to bypass privacy regulations^13^. In this article, we address such criticisms by highlighting existing regulatory guidance from privacy authorities and examining how these regulators approach SDG and synthetic data around the world. We conducted a review of regulatory guidelines related to synthetic data provided by privacy authorities. To date, only three jurisdictions, namely the United Kingdom (UK), South Korea, and Singapore, have published specific guidelines on synthetic data. Our aim is to describe contemporary regulatory perspectives on SDG and synthetic health data, to identify areas of convergence and divergence across the selected frameworks and to understand the conditions under which synthetic data may be considered as non-personal information that is safe to share and disseminate with relatively few obligations.

While we are still in the early days of regulation, this manuscript offers the first comparative analysis of regulatory guidelines on this topic. It highlights the concerns and risks that are deemed important to address, and how regulators and legislators have approached them thus far. These insights are relevant not only for those actively shaping the field, but also for the broader research community that will increasingly generate and use synthetic data in practice.

## Synthetic Data for Healthcare

### The potential

The growing interest in SDG is particularly pronounced in the healthcare sector and digital medicine, where the volume, sensitivity, and regulatory complexity pose challenges to data use and access for secondary purposes, such as research^14^. To highlight this challenge, an examination of the success rates of getting individual-level data for research projects from authors found that the percentage of the time these efforts were successful varied significantly and was generally low at 58^15^, 46^16^, 25^17^, 14^18^, and 0%^19^.

SDG holds the potential of unlocking healthcare data for secondary research, and accelerating innovation^20–22^. Several initiatives have already made synthetic versions of their healthcare data broadly available, such as the National COVID Cohort Collaborative by the National Institutes of Health of the United States (US)^23^, the Medicare Claims Synthetic Public Use Files in the US^24^, the synthetic cardiovascular cohort and the synthetic COVID-19 dataset provided by the Clinical Practice Research Datalink team in the UK^25,26^, synthetic cancer data from Public Health England^27^, and synthetic microdata from Israel’s National Registry of Live Births^28^. National initiatives like SynD in Australia^29^, Europe’s SYNTHIA^30^ or the NFDI4Health in Germany^31^ are supporting the responsible adoption of synthetic health data. Recently, study authors have been making synthetic variants of data used in their research papers publicly available to enable open science^28,32–35^. Furthermore, there are examples of health research studies using synthetic data not requiring ethics approval because the data was considered as not containing actual patient information^36,37^.

**Fig. 1:**
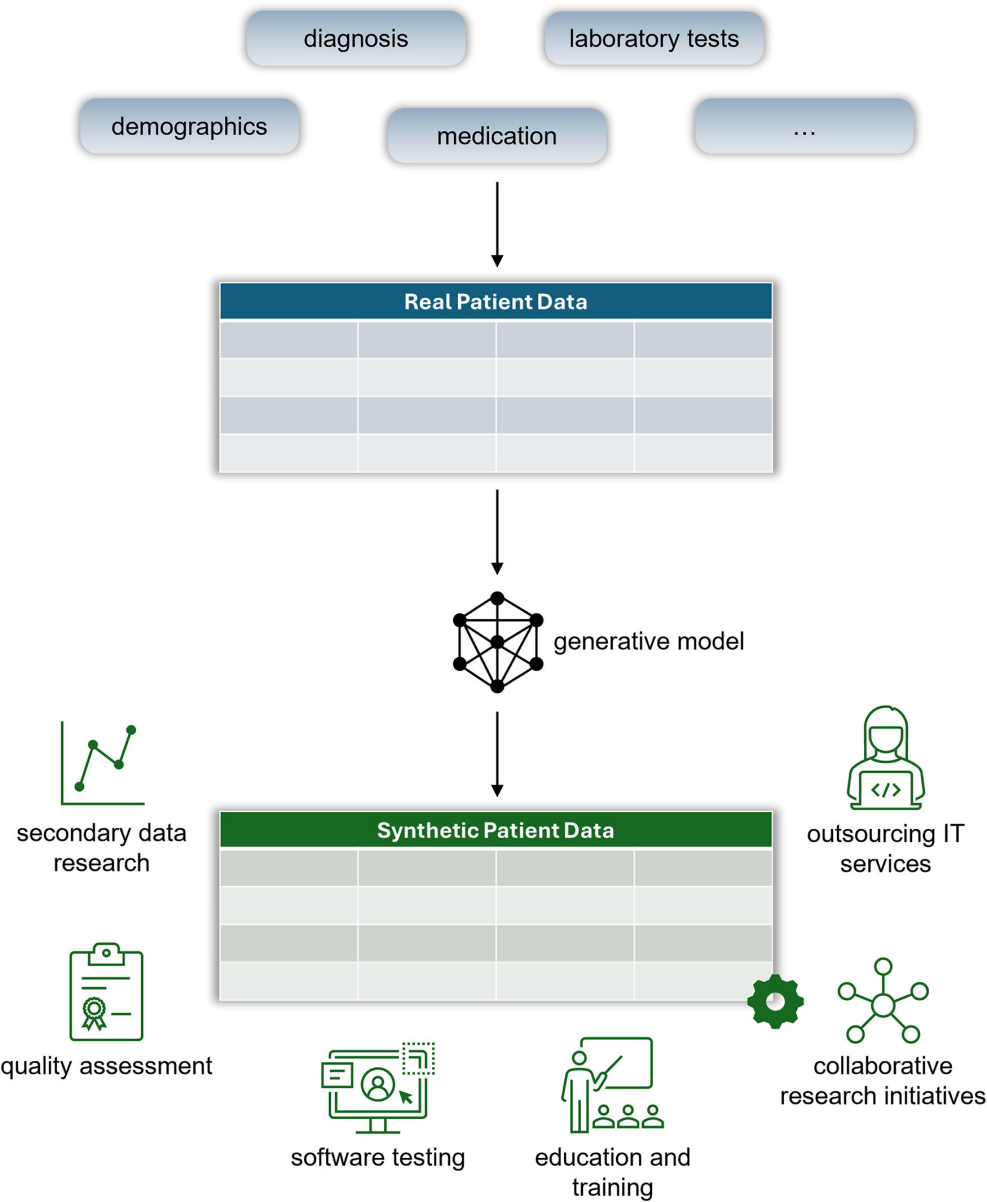
Tabular Synthetic Data Generation for Healthcare. There are multiple generative models that can be leveraged including AI/ML^20^.

Synthetic data also offers a privacy-preserving way to develop, test and validate software required for the digital health infrastructure without exposing personal health information^21^. This includes AI/ML-based software that is increasingly entering the healthcare sector^22,38–40^. Finally, synthetic data holds value for education and training by providing realistic yet synthetic datasets^41^. Figure 1 illustrates the potential of tabular SDG with exemplary use cases.

### The concerns and risks

While these examples illustrate the potential of SDG in the healthcare sector, some authors have also raised valid concerns regarding the potential bias of synthetic data and the risk of blindly trusting synthetic data as being free of disclosure risk^13,42,43^. These concerns are described below.

Bias has been a long-standing concern in biomedicine as it can be inherent in study design, clinical decisions, and healthcare access^44,45^, ultimately contributing to discriminatory decisions. For example, selection bias in clinical trials combined with treatment effect heterogeneity results in external validity bias^44,46,47^ which, in turn, can substantially aggravate health disparities^47,48^.

Ethical concerns around bias and discriminatory decisions are typically not intrinsic to SDG or synthetic data itself but are more broadly related to the use of AI/ML. The problem as expressed in the Impact Assessment of the regulation on artificial intelligence by the European Commission is the “functional dependence [of AI] on data and the quality of data”, as biases in the training data can be reproduced and even reinforced by AI models potentially resulting in discriminatory decisions^49^. The potential for bias in AI/ML has been widely acknowledged and actively researched. In recent years, the community has come up with ethical AI guidelines^50–52^, and biases are also addressed in SDG-specific guidelines, such as the United Nations University policy guidelines^53^. This means that principles, such as responsibility, non-maleficence, privacy, transparency, and justice and fairness do apply to SDG^50,53^.

Privacy concerns in SDG arise from personal health information being used during model training, and residual privacy risks that may persist in the resulting synthetic data. SDG can, as shown in Fig. 1, reduce reliance on personal health information, thereby mitigating disclosure risks (for the relevant terminology, see Table 1). It is designed to enhance privacy, should protect against identity disclosure *by design* and is therefore typically regarded as providing higher privacy protection than more traditional anonymization techniques^54^. But the disclosure of personal information can also happen, for example, when the SDG model overfits and through inference^8,55–57^.

**Table 1:** Key Terminology on Privacy in This Manuscript

**Disclosure**
We refer to disclosure as the act of revealing personal information from a dataset. This is in line with the definition by the ISO/IEC standard on Information security, cybersecurity and privacy protection – Privacy enhancing data de-identification framework^101^. Personal information can also be revealed from the SDG model itself. This is, however, not the focus of this article.
**Identity disclosure**
Identity disclosure is when an identity can be correctly assigned to a record in a dataset. It is closely related to the concepts of singling out and linkability^101,107^
**Personal information**
The legal terminology around personal information varies across jurisdictions^100^. In this manuscript, we use the term personal information to refer to any information that relates to an identified or identifiable individual and that therefore falls within the scope of privacy and data protection regulations. Depending on the jurisdiction, equivalent terms include personal data (e.g., Europe) or personally identifiable information (e.g., United States of America (USA))^108^. The concept of non-personal information is consequently also tied to the jurisdiction under investigation but typically allows for a very low residual risk^108^.
**Privacy**
In this manuscript, the term privacy is used to refer to whether personal information is disclosed, rather than the potential harm that may result from such disclosure. This is sometimes also referred to as informational privacy^108^. Our focus of privacy is on the individual as a data subject and does not extend to broader interpretations, such as group or collective privacy (see e.g., refs. ^109,110^.). These broader concepts focus more on downstream harm and are typically not included under more technical definitions of privacy. However, they remain relevant in the context of responsible data use and disclosure and are explicitly noted throughout the manuscript when taken into consideration.
**Privacy metric**
A privacy metric measures privacy vulnerability in synthetic data^87^. A metric is typically defined by its underlying assumptions (e.g., about the adversary), the methodological design (such as how an attack is mimicked by the data custodian), and the vulnerability reporting (e.g., the performance measurements).
**Privacy risk and vulnerability**
Privacy risk can be characterized by two components: the privacy vulnerability as a characteristic of the data, which is a measure of the likelihood of a privacy violation as a characteristic of the data^87,111^, and the contextual risk, such as organizational and technical safeguards, which accounts for the likelihood of an attack^112^. This is sometimes also referred to as threat modeling^101^. The contextual notion of privacy risk means that privacy evaluation is tied to the specific situation in which it is conducted.

## The Regulation of SDG

Given the privacy concerns related to SDG, it is relevant to understand how SDG and synthetic data are regulated, as regulatory frameworks are intended to mitigate the very risks that come with the technologies they regulate. Legal analyses are an important part of interpreting current regulations with respect to evolving technologies, such as SDG. For example, under the European General Data Protection Regulation (GDPR), the UK GDPR or the Canadian Personal Information Protection and Electronic Documents Act, opinions typically treat SDG as another (novel) PET^58–64^. In this context, the process of SDG is generally seen as processing personal data, requiring a lawful basis under applicable privacy law, while resulting synthetic data may qualify as non-personal data if residual risks are deemed sufficiently remote. While some authors have argued that such a treatment may be too conservative and have proposed to move beyond the traditional PET regulation^62^, they also acknowledge that treating SDG as a PET remains the most likely legal standard in practice. This suggests that SDG requires the same rigorous considerations as other PETs, such as anonymization, in regulatory practice and cannot simply bypass privacy and data protection regulations by default.

SDG that leverages AI for model training would further fall under the scope of the European AI Act where obligations are commensurate with the level of risk. These include, for example, bias examination in training data for high-risk AI, which SDG in the healthcare sector may be subject to ref. ^65^. Other AI legislation, such as the Utah AI Policy Act^66^, clarifies accountability for consumer AI interactions and, while not the focus of the law, explicitly excludes synthetic data from the definition of personal data, provided it is not “linked or reasonably linkable to an identified individual or an identifiable individual”.

Data protection authorities have also started to publish explicit SDG guidelines that give valuable insights into how regulators approach SDG: the 2023 published guidance on privacy-enhancing technologies by the Information Commissioner’s Office (ICO) in the UK^67^, the 2024 guide from the Personal Data Protection Commission (PDPC) in Singapore^68^, and the 2024 guide from the Personal Information Protection Commission (PIPC) in South Korea^69^.

Other (data) authorities have also expressed initial thoughts on the privacy-protective potential of SDG in webinars, reports or blogposts (see e.g. ref. ^62^ for an overview in the European context, the federal privacy commissioner blogpost^70^ and the consultation session on the Ontario de-identification guidance draft^71^ as examples in Canada, and the sections on synthetic data in the Guidelines for Evaluating Differential Privacy Guarantees by the US National Institute of Standards and Technology^72^).

It is, however, important to distinguish these from formal regulatory guidelines. Guidelines represent authoritative documents that not only reflect legal interpretations but also channel regulatory recommendations and expectations. For example, the UK ICO guidance is the result of a structured, multi-phase process, consistent with the ICO’s internal guidance development framework^73^, and includes the following development stages: (1) Drafting, (2) Public consultation or call for views (for the PET guidance this occurred in 2022^74^), (3) Redrafting (following consultation) and (4) Publication on the website. As with other regulatory guidelines, the ICO guidance is not legally binding in the same sense as statute, but if an enforcement action were taken against an organization undertaking processing activities for which PETs may have been appropriate, this guidance would serve as a relevant and authoritative reference in assessing whether PETs were properly implemented or should have been implemented.

These three guidelines are key to the adoption of SDG as they increase regulatory clarity and create precedents. They also provide insights into the current understanding of synthetic data across its lifecycle by regulatory authorities. These guidelines are examined below in more detail to present a realistic picture of how SDG and its associated risks are actually regulated.

From the perspective of the three regulators, the healthcare sector is a typical use case for SDG. For example, the ICO published a case study on synthetic data generated from prescription databases to develop and test a system for planning public services^75^. In South Korea, prior to the publication of the synthetic data guidelines, the PIPC released five synthetic reference datasets. Two of these datasets involve healthcare settings: one dataset containing tabular data on individuals’ blood sugar levels along with data on measurement time, age, and time lapsed from food intake, and the other dataset containing teeth image data with or without cavities^76^. Similarly, two of the four case studies provided in the Singapore guidelines are from the healthcare sector: one case study where SDG is implemented instead of anonymization by a pharmaceutical company to provide access to higher utility health data for a researcher, and another one where synthetic data is used to preview health data, thereby facilitating scientific collaboration^68^.

These examples underscore the relevance of the regulatory guidelines to the healthcare sector and to the central question of this manuscript: is synthetic data protective of patient privacy? We analyze three questions and present the regulatory logic found across the guidelines (see Table 2). Similar questions have been posed in legal analyses around SDG^6,58–61^ and these questions are essential for understanding the risks that must be mitigated in synthetic data, as well as the conditions under which synthetic data can be considered as non-personal information and thereby protective of patient privacy.

**Table 2:** Summary of Regulatory Perspectives on SDG and Synthetic Data

ICO, UK^67^	PDPC, Singapore^68^	PIPC, South Korea^69^
**Can personal information be processed without consent for SDG (i.e., training SDG models on real individual-level data without additional consent)?**		
Yes, if there is another lawful basis to use personal information for SDG, such as legitimate interest.		Yes, SDG is a permitted use of personal information where no separate consent is required.
**Are there conditions for treating synthetic data as non-personal information?**		
Yes, conditions apply for synthetic data to be treated as non-personal information. Privacy evaluation is consequently an essential part of good SDG practice. Precise criteria (i.e., metrics and thresholds) remain an active field of research.		
**What are other recommended evaluation practices beyond privacy?**		
Quality assessment, bias detection	Quality assessment, sensitive or misinformative insights from synthetic data	Quality assessment, bias and falsified data

### Can personal information be processed without consent for SDG (i.e., training SDG models on real individual-level data without additional consent)?

The response to this question is jurisdiction-dependent and depends on the applicable lawful bases for processing personal information, as set forth in the respective regulatory framework. While these vary, all three regulatory guidelines^67–69^ highlight that whenever personal information is used to train the SDG model, this can be seen as a form of processing of personal data. As such, the act of SDG itself falls within the scope of privacy and data protection regulation, regardless of whether consent is the specific legal basis applied.

In the UK, there are six (equally valid) lawful bases: consent, contract, legal obligation, vital interests, public task and legitimate interests^77^. These lawful bases are consistent with those outlined in the recently enacted Data Use and Access Act^78^. The Act introduces recognized legitimate interests as a clarification and an extension to the existing lawful basis of legitimate interests. This is a predefined list of purposes for which the legitimate interest in processing personal information is easier to justify and would not require a legitimate interest assessment. The most appropriate lawful basis for SDG depends on the circumstances of SDG. For example, the lawful basis for SDG could be a legitimate interest in using synthetic data for scientific purposes. Scientific research under the Data Use and Access Act^78^ is defined as “any research that can reasonably be described as scientific, whether publicly or privately funded and whether carried out as a commercial or non-commercial activity”. In fact, the National Health Service Research Authority already clarified in 2018 (prior to Brexit) in their guidance on the European GDPR that public task or legitimate interests are typically relied upon by research organizations^79^.

In most cases, it is also likely that the processing of personal information for SDG is compatible with the original purpose of collection. The ICO guidance on anonymization supports this position, stating that compatibility with the original purpose is very likely the case for many situations when anonymizing data^80^.

For example, if data originally collected for a public health research purpose were used to generate a synthetic dataset to help researchers prototype their analyses before accessing the real data, this would very likely be considered compatible with the original purpose. However, if this data were instead used to generate synthetic data for unrelated commercial uses, such as identifying customer characteristics for marketing campaigns, this could raise questions about purpose compatibility and, consequently, concerns about the lawful basis and the fairness of SDG. A lawful basis, compliance with the fairness principle and purpose compatibility are required under UK GDPR Article 6, Article 5 and Recital 50, so that the unrelated use of the synthetic data in this example would then need to be evaluated to determine whether the SDG is compatible with the original purpose of collecting the data, and therefore remains a lawful processing of personal information. Commercial uses, such as identifying customer characteristics for marketing campaigns, are not set out in the annex of the Data Use and Access Act as a potential compatible use^78^, so that in this case, it would be treated as a new purpose requiring a respective lawful basis, such as legitimate interest^78^ with a respective legitimate interest assessment. In such a legitimate interest assessment, the use of synthetic data would very likely tilt the balance towards reducing the risk to individuals.

While legitimate interest may serve as a lawful basis for processing personal data for SDG in this example, this approach might not align with recommendations expressed in the ICO’s guidance on anonymization. These highlight that “you should only use anonymous information in ways people would reasonably expect; consider whether people would reasonably expect you to retain the data in identifiable form; and assess whether turning personal data into anonymous information affects people. For example, if you are using the anonymous information to make decisions or decide how you treat people, and how you can justify any adverse impact”^80^. Even though this is formulated as a recommendation or best practice rather than a statutory obligation (“should” versus “must”), such provisions are relied upon in regulatory decisions and inform how regulators interpret legally binding principles, such as fairness, in practice. Organizations that disregard such recommendations expose themselves to regulatory scrutiny and potential enforcement action, a process that can result in corrective measures, but also in loss of trust and reputational damage, even if practices remain strictly speaking lawful. This means that even if the unrelated commercial use based on legitimate interests in this example meets the criterion of a lawful basis, it may fall short of good practice from the perspective of the ICO’s guidance on anonymization.

In Singapore, while consent is a legal basis for the use of personal information for SDG, there are also exceptions to the obligations, such as the legitimate interest exception or where SDG is for research purposes. In South Korea, SDG is a permitted use of personal information where no separate consent is required for SDG, although further legal considerations may be needed depending on the nature of the synthetic data generated. For example, when using healthcare data for SDG, the Bioethics and Biosafety Act may apply, and a research ethics board review could be required when used for research.

This means that, in the jurisdictions under consideration, there are situations where personal information can be processed for SDG without additional consent. However, in other jurisdictions, the situation may be similar to that under Canada’s provincial health law from Ontario (i.e., Personal Health Information Protection Act)^81^ where SDG would be covered by the permitted uses of personal information without consent, or it may not be directly addressed, such as under Canada’s federal privacy law (i.e., Personal Information Protection and Electronic Documents Act)^82^.

### Are there conditions for treating synthetic data as non-personal information?

All three guidelines acknowledge that there are conditions for treating synthetic data as non-personal information. They also acknowledge that the definition of these conditions (i.e., metrics and thresholds) remains an active field of research where precise criteria were lacking at the time of writing the guidelines.

According to the ICO guidance^67^, the question of whether synthetic data can be processed as non-personal information depends on “whether the personal information on which you model the synthetic data can be inferred from the synthetic data itself”. This is further complemented by the UK data protection law (recital 26 of UK GDPR), where all means should be considered that are “reasonably likely to be used, such as singling out”. Similar requirements are expressed in the Singapore and South Korea guidelines. If, and only if, the residual risks are very low, then synthetic data can be treated as non-personal information and falls outside the scope of privacy and data protection regulations. This conditional assessment aligns with the academic literature, where some studies have demonstrated privacy vulnerabilities in synthetic data (e.g., refs. ^83,84^,), while others have not (e.g., refs. ^85,86^,).

Following this reasoning, privacy and data protection is one of the evaluation dimensions mentioned in all three guidelines. The strength of recommendation, however, varies slightly: while the ICO guidance requires such evaluation (“requires assessment” and “must be subjected to”)^67^, the South Korea guidelines recommend it^69^. Similarly, the Singapore guidelines, as a whole, are general good practice recommendations and not considered a requirement.

The guidelines do not present a prescribed set of vulnerability metrics but recommend exemplary measurements and/or practices to mitigate vulnerability. The ICO guidance, for example, mentions that outlier protection in synthetic data could be done via suppression or differential privacy^67^. It is important to mention that there is no definition of outlier provided and that definitions of outliers in the context of privacy in synthetic data vary across the academic literature^87^, so that this approach would require further clarification to support consistent and effective implementation in practice. In the Singapore guidelines, examples are provided to quantify attribute and membership disclosure vulnerability, and again, outlier removal is mentioned as a way to mitigate disclosure risk alongside data minimization and generalization during the SDG process^68^. The idea in the Singapore guidelines was to focus on vulnerability mitigation throughout the SDG process and controlling residual risk through governance controls, contractual processes, and technical measures. The South Korean guidelines present exemplar metrics for singling out, linkability and inference, and the same practices to mitigate disclosure risks as in the other two guidelines are mentioned^69^. The South Korean guidelines also contain various measurement metrics that can be used when evaluating vulnerability and suggest an evaluation process under which a review panel of 3 (or more) experts would make an assessment as to whether SDG was done properly and whether the resulting synthetic data can be deemed non-personal.

As mentioned previously, all three regulatory authorities acknowledge that privacy and data protection are active fields of research in synthetic data. Consequently, specific thresholds for a sufficiently low disclosure risk level are not (or only to a limited extent) provided. In the Singapore guidelines, thresholds are explicitly discussed and provided for identity disclosure risk from international guidelines for anonymized data, with the caveat that risk estimation varies between synthetic and anonymized data^68^. The South Korean guidelines give more guidance in the context of thresholds by providing a preferred absolute threshold value for their example linkability metric, as well as a way to calculate thresholds derived from a baseline disclosure that is deemed to be acceptable, but again, “more extensive research is needed”^69^.

### What are other recommended evaluation practices beyond privacy?

All three regulatory guidelines consider evaluations beyond privacy as part of risk mitigation in synthetic data. This includes the quality of the data as well as further ethical considerations.

Quality evaluation is generally an assessment of whether “the synthetic data you use is an accurate proxy for the original data”^67^. In the academic literature, multiple metrics have been proposed to evaluate the quality of synthetic data. They are typically grouped into metrics of fidelity (or broad utility) and of downstream utility (or narrow utility)^8,57^. Fidelity is concerned about the similarity between synthetic and real data with respect to their statistical properties and distributions. Utility metrics evaluate how well synthetic data performs downstream tasks, such as AI/ML modeling or statistical inference. The choice of fidelity and downstream utility metrics ultimately depends on the use case, and the regulatory guidelines provide non-prescriptive examples of such metrics.

Further ethical considerations are mentioned in all three guidelines. The South Korean guidelines, for example, acknowledge the risk of bias and falsified data^69^. The ICO guidance explicitly recommends that bias detection and correction in SDG is ensured and demands it as mandatory if “you are using synthetic data to make decisions that have consequences for people (i.e., legal or health consequences)”^67^. And in the Singapore guidelines, the evaluation includes whether or not insights from synthetic data are sensitive or misinformative, and it is highlighted that SDG can replicate trends of source data that may be sensitive^68^.

These considerations emphasize that synthetic data can reproduce not only valuable but also sensitive patterns. This concern is sometimes expressed in the context of synthetic data, but in fact, it reflects a broader concern with any data-driven approach: high utility enables meaningful insights, but can also expose group-level patterns that may lead to collective downstream harm. This means that beyond individual privacy, if certain groups can be negatively affected by the use of synthetic data—whether through the nature of the data itself (i.e., bias) or through the purpose behind data use—their collective harm must also be considered.

### Limitations

The analysis above is limited to tabular data. While multiple considerations within this scope are relevant for synthetic text, image, and video data as well, further analysis is needed for these alternative modalities. The South Korean guidelines cover image data as well as tabular data, and the ICO has published work on synthetic media^88^, but nonetheless, further research is warranted to extend our analysis to other data modalities.

We also did not focus on the SDG models themselves in this article, but the ICO guidance and the Singapore guidelines acknowledge that there may be different risks in models than in the synthetic datasets. In the ICO guidance, the vulnerability of SDG models to model inversion, or membership disclosure and attribute disclosure, is mentioned^67^, in the Singapore guidelines their vulnerability towards reconstruction is highlighted^68^ and the South Korean guidelines contain a short summary on the strengths and vulnerabilities of different types of SDG models^69^. In general, when not critical, the generative model should not be disclosed, and this is consistent with current practices. If disclosure is necessary, a thorough risk assessment must be conducted.

Ultimately, only three regulatory bodies have published explicit guidelines that are directly applicable to SDG. Given the importance of the topic, more regulators around the world may produce their own guidelines on this topic in the future and they may reach different conclusions.

## Discussion

This article provides a contemporary picture of how regulatory authorities understand synthetic (health) data across its lifecycle. We argue that regulatory guidelines encompass the management of privacy, data protection, quality, and bias risks in synthetic data. Claiming that there is a lack of oversight in SDG does not accurately reflect the current state of practice. Our analysis deconstructs the narratives that present SDG as inherently a threat to privacy, data protection and ethical principles: those who use or regulate SDG acknowledge the promising potential of SDG as a PET but are well aware of potential bias and discriminatory decisions, as well as privacy and data protection considerations. The expectation is that these aspects would be properly evaluated and mitigated when SDG is applied.

Privacy and data protection considerations encompass the lawful basis required when processing (or using) personal information to train SDG models, as well as the regulation of the synthetic data itself. Synthetic data can be considered as non-personal information if residual disclosure risks are very low. And while an international standard on evaluating the residual risk in synthetic data with corresponding thresholds has not yet been developed, it is not questioned that such an assessment is necessary. Guidelines also make clear that utility, bias and fairness are relevant parts when evaluating synthetic data.

These findings suggest that SDG is not a means of circumventing privacy and data protection regulations. Like other PETs, it is subject to such regulation whenever personal information is involved, and the resulting data are only outside its scope if it can be considered as non-personal information.

Concerns about SDG as a way to bypass privacy regulation have mainly focused on commercial actors^13^ and it is important to note that concerns about the use of health data by commercial entities are not unique to synthetic data. More broadly, in the context of health data sharing, there is well-documented and persistent distrust by patients toward commercial actors, in particular pharmaceutical and insurance companies^89–94^ and recent publications reflect a public discomfort about the commercialization of anonymized health data in the primary care sector^95,96^. At the same time, studies suggest that such concerns are not universal^91,93^. While some individuals express distrust towards commercial actors, others recognize potential societal benefits from private-sector-driven innovations. Nonetheless, there have been cases where commercial actors have used data without sufficient transparency, thereby reinforcing these concerns and prompting regulatory responses.

For example, the Ontario’s Information and Privacy Commissioner investigated a case where a company providing electronic health record software de-identified personal health information and sold it to a third party^97^. The investigations had negative findings about transparency, but there was no evidence that the data had not been properly anonymized or that any unlawful use of personal health information had occurred. A similar conclusion was reached by the federal Office of the Privacy Commissioner in Canada in its investigation about the sharing of anonymized mobility data between a mobile network operator and the Public Health Agency of Canada in the context of COVID-19 surveillance^98^. In this example, again, negative findings about transparency were reported, but the anonymization practices were deemed adequate and data use and disclosure consistent with applicable privacy law.

This demonstrates two things. First, in the regulatory context of these examples, when anonymization was properly conducted and residual risks are very low, regulatory authorities have found the use and disclosure of anonymized health data to be consistent with existing privacy laws. At the same time, these cases show that even if data use and disclosure practices are lawful, corrective measures can still be necessary where divergences from best practice are identified, reflecting the regulatory weight of best practice recommendations. Second, it also highlights persistent public ambivalence toward commercial actors^91–93^: What is deemed lawful within applicable privacy law may still be perceived as controversial by the public. As in our example, sharing de-identified data for commercial purposes is not inherently problematic from a regulatory perspective, if carried out in line with respective privacy law, yet such practices can produce negative public reactions. This suggests that legal compliance alone is insufficient to maintain public trust and maintain the social license for the secondary use and disclosure of health information^99^.

There are several approaches to bridge this gap, such as strengthening transparency requirements, providing a more enforceable set of guidelines^64^ or complementing privacy regulation with an additional layer of ethical oversight, such as is already done in the context of research ethics boards^82^. Another approach would be to bring anonymized data back within the scope of privacy law^10^. This may, however, undermine incentives for the adoption of PETs and could create additional burdens for secondary data use. Ultimately, these controversies are not specific to synthetic data, but part of a broader discussion about how to govern the secondary use of health data in ways that are both legally sound and socially acceptable.

Also, our findings should not be taken to imply that the regulation of SDG is perfectly clear. Most existing privacy and data protection laws, for example, are designed to be technology agnostic and SDG or synthetic data is not explicitly mentioned in the statutes. Also, definitions about identifiability are vague and differ across jurisdictions (see e.g., G7 initiative on harmonizing terminology^100^), and while it is a common understanding that non-identifiability allows for a very low residual disclosure risk and case law relevant to anonymization could be leveraged for SDG as well, actual court cases providing precedents specifically about synthetic data are lacking. The presented analysis, however, shows that SDG can be treated as another form of PET, for which there is existing experience, judicial precedents, and clear regulation.

In ref. ^62^, criticism of such treatment has been raised with concerns about its feasibility, particularly about the potential reduction in data utility and the usage restrictions resulting from an overly precautionary approach, the high burden of demonstrating very low residual disclosure risks, and the practical challenges in applying data subject rights if synthetic data remains personal information. The main challenge, however, may lie less in the approach itself and more in the fact that standards developed for anonymization (e.g., ISO/IEC 27559^101^) focus primarily on identity disclosure metrics and that these considerations are not directly transferable or applicable to SDG. Consequently, the landscape of privacy metrics for synthetic health data has been fragmented, with no consensus on which metrics to use^8,102^. Therefore, there is a strong imperative to develop standards for SDG^103^. Notably, recently privacy experts have collaborated to reach consensus in measuring disclosure vulnerability in synthetic data^87^ and initiatives to develop new standards for SDG, such as the drafted ISO/IEC AWI TR 42103 (Information technology—Artificial intelligence—Overview of synthetic data in the context of AI systems), or the Synthetic Data Industry Connections activity under the IEEE Standards Association have been formed^104,105^.

Regulatory sandboxes can further promote the adoption of and trust in SDG by providing a testing ground for participants to pilot innovative approaches, with technology, financial and regulatory support^106^. They require mutual engagement from both the regulator and the participant to find a solution to a challenging regulatory situation. While regulatory sandboxes are not solely aimed at PETs, SDG is a good candidate given its rapidly evolving nature and innovative character. Insights and learnings from such sandbox use cases can then be considered in subsequent guidelines.

## Data Availability

No datasets were generated or analyzed during the current study.
